# Supplementary material for: From sequence to enzyme mechanism using multi-label machine learning
Source: BMC Bioinformatics. 2014 May 19;15:150. doi: 10.1186/1471-2105-15-150 (PMC4229970; doi:10.1186/1471-2105-15-150)
Supplement: Additional file 2 — Java code of ml2db. Additional file ml2db_code.tar.gz contains the Java source code to run the multi-label machine learning experiments and save the results to database. The code’s Javadoc is included. [file 1471-2105-15-150-S2.zip › additional file 2/ml2db/ml2db/doc/mulan/classifier/transformation/EnsembleOfPrunedSets.html]

EnsembleOfPrunedSets


JavaScript is disabled on your browser.


- Overview
- Package
- Class
- Use
- Tree
- Deprecated
- Index
- Help

- Prev Class
- Next Class

- Frames
- No Frames

- All Classes

- Summary:
- Nested |
- Field |
- Constr |
- Method

- Detail:
- Field |
- Constr |
- Method


mulan.classifier.transformation

## Class EnsembleOfPrunedSets

- java.lang.Object
- - mulan.classifier.MultiLabelLearnerBase
  - - mulan.classifier.transformation.TransformationBasedMultiLabelLearner
    - - mulan.classifier.transformation.EnsembleOfPrunedSets

- All Implemented Interfaces:
  :   java.io.Serializable, MultiLabelLearner, weka.core.TechnicalInformationHandler

  ---

    

  ```
  public class EnsembleOfPrunedSets
  extends TransformationBasedMultiLabelLearner
  ```

  Class implementing the Ensemble of Pruned Sets algorithm(EPS) . For more information, see

  Version:
  :   2012.02.27

  Author:
  :   Emmanouela Stachtiari, Grigorios Tsoumakas

  See Also:
  :   Serialized Form

- - ### Constructor Summary

    Constructors

    Constructor and Description

    `EnsembleOfPrunedSets()`

    Creates a new instance with default values

    `EnsembleOfPrunedSets(double aPercentage,
    int aNumOfModels,
    double aThreshold,
    int aP,
    PrunedSets.Strategy aStrategy,
    int aB,
    weka.classifiers.Classifier baselearner)`
  - ### Method Summary

    Methods

    | Modifier and Type | Method and Description |
    | --- | --- |
    | `weka.core.TechnicalInformation` | `getTechnicalInformation()` Returns an instance of a TechnicalInformation object, containing detailed information about the technical background of this class, e.g., paper reference or book this class is based on. |
    | `java.lang.String` | `globalInfo()` Returns a string describing classifier |

    - ### Methods inherited from class mulan.classifier.transformation.TransformationBasedMultiLabelLearner

      `getBaseClassifier`
    - ### Methods inherited from class mulan.classifier.MultiLabelLearnerBase

      `build, getDebug, isUpdatable, makeCopy, makePrediction, setDebug`
    - ### Methods inherited from class java.lang.Object

      `equals, getClass, hashCode, notify, notifyAll, toString, wait, wait, wait`

- - ### Constructor Detail


    - #### EnsembleOfPrunedSets

      ```
      public EnsembleOfPrunedSets()
      ```

      Creates a new instance with default values


    - #### EnsembleOfPrunedSets

      ```
      public EnsembleOfPrunedSets(double aPercentage,
                          int aNumOfModels,
                          double aThreshold,
                          int aP,
                          PrunedSets.Strategy aStrategy,
                          int aB,
                          weka.classifiers.Classifier baselearner)
      ```

      Parameters:
      :   `aNumOfModels` - the number of models in the ensemble
      :   `aStrategy` - pruned sets strategy
      :   `aPercentage` - percentage of data to sample
      :   `aP` - pruned sets parameter p
      :   `aB` - pruned sets parameter b
      :   `baselearner` - the base learner
      :   `aThreshold` - the threshold for producing bipartitions
  - ### Method Detail


    - #### getTechnicalInformation

      ```
      public weka.core.TechnicalInformation getTechnicalInformation()
      ```

      **Description copied from class: `TransformationBasedMultiLabelLearner`**

      Returns an instance of a TechnicalInformation object, containing detailed
      information about the technical background of this class, e.g., paper
      reference or book this class is based on.

      **Specified by:**
      :   `getTechnicalInformation` in interface `weka.core.TechnicalInformationHandler`

      **Overrides:**
      :   `getTechnicalInformation` in class `TransformationBasedMultiLabelLearner`

      Returns:
      :   the technical information about this class


    - #### globalInfo

      ```
      public java.lang.String globalInfo()
      ```

      Returns a string describing classifier

      **Overrides:**
      :   `globalInfo` in class `TransformationBasedMultiLabelLearner`

      Returns:
      :   a description suitable for displaying


- Overview
- Package
- Class
- Use
- Tree
- Deprecated
- Index
- Help

- Prev Class
- Next Class

- Frames
- No Frames

- All Classes

- Summary:
- Nested |
- Field |
- Constr |
- Method

- Detail:
- Field |
- Constr |
- Method
